# Routine biochemical screening on admission saves neither money nor time: a randomised trial

**DOI:** 10.1186/1757-7241-20-S2-P44

**Published:** 2012-04-16

**Authors:** Manan Pareek, Felix Haidl, Lars Folkestad, Mikkel Brabrand

**Affiliations:** 1 Akut Medicinsk Modtageafsnit, Sydvestjysk Sygehus, Esbjerg, Denmark; 2 Medicinsk Afdeling, Sygehus Lillebælt, Vejle, Denmark

## Background

Evidence justifying the use of routine laboratory screening on admission is weak. The principle, however, has been widely used for decades, the arguments being convenience, a more rapid and thorough screening, and thus reduced admission time. This study investigated the differences in cost of tests and admission time if doctors at admission prescribed single biochemical tests rather than using predefined test panels.

## Methods

191 consecutive patients admitted at an adult acute medical admissions unit were randomly assigned to either predefined blood tests (n = 100) or blood tests ordered by the admitting doctor after history taking and physical examination (n = 91). Data was assumed non-normal distributed and reported descriptively as median [interquartile range]. Between group differences were calculated using the Wilcoxon rank sum test and Fischer’s exact test with a two-tailed alpha of 0.05, beta 0.20. The trial was registered at http://www.clinicaltrials.gov, no. NCT00768547.

## Results

The median number of tests performed per patient was 17 in both groups (IQR [14-22] in the routine group, [12-21] in the doctor group, (p = 0.30)). Cost per patient was non-significantly higher in the routine group (618 DKK, [493-803] vs. 564 DKK, [434-812], (p = 0.19)). Furthermore, there was a non-significant (p = 0.08) trend towards increasing admission time when routine tests were performed (median 4 days, [2-6] vs. 2 days, [2-6]).

## Conclusion

Our results showed no significant difference in either number of ordered tests, total cost or admission time between the two groups. This concurs with the literature where studies have failed to show a positive impact of routine admission testing on patient care, length of stay and hospital costs.

